# Treatment of osteoarthritic knee with high tibial osteotomy and allogeneic human umbilical cord blood–derived mesenchymal stem cells combined with hyaluronate hydrogel composite

**DOI:** 10.1186/s13287-025-04356-9

**Published:** 2025-04-28

**Authors:** Bo Seung Bae, Jae Woong Jung, Gyeong Ok Jo, Seon Ae Kim, Eun Jeong Go, Mi-La Cho, Asode Ananthram Shetty, Seok Jung Kim

**Affiliations:** 1 Department of Orthopaedic Surgery, College of Medicine, The Catholic University of Korea, Seoul, Republic of Korea; 2 The Rheumatism Research Center, Catholic Research Institute of Medical Science, College of Medicine, The Catholic University of Korea, Seoul, Republic of Korea; 3 Institute of Medical Sciences, Faculty of Health and Social Care, Canterbury Christ Church University, Canterbury, UK; 4 Department of Orthopedic Surgery, Uijeongbu St. Mary’s Hospital, College of Medicine, The Catholic University of Korea, 271, Cheonbo-ro, Uijeongbu-si, Gyeonggi-do Republic of Korea

**Keywords:** Cartilage regeneration, High tibial osteotomy, Mesenchymal stem cell, Osteoarthritis

## Abstract

**Background:**

Delaying total knee arthroplasty is crucial for middle-aged patients with severe osteoarthritis. The long-term outcomes of high tibial osteotomy (HTO) remain uncertain. Recently, mesenchymal stem cells (MSCs) have shown promising potential in enhancing cartilage regeneration. Therefore, this study aimed to assess cartilage regeneration following the implantation of allogeneic human umbilical cord blood–derived mesenchymal stem cells (hUCB-MSCs) with HTO.

**Methods:**

In this case series, ten patients underwent hUCB-MSC implantation with HTO. The median age was 58.50 (range: 57.00–60.00) years, and the mean body mass index was 27.81 (range: 24.42–32.24) kg/m^2^. Clinical outcomes, including the Western Ontario and McMaster Universities Osteoarthritis Index (WOMAC), visual analog scale (VAS), Physical Component Score (PCS) and Mental Component Score (MCS) from the 36-Item Short-Form Health Survey (SF-36), were evaluated 6 months, 1 year, and 2 years postoperatively. Cartilage status of the medial femoral condyle (MFC) was assessed during hardware removal surgery, at least 2 years after the initial procedure, and compared with preoperative MFC cartilage status regarding lesion size and International Cartilage Repair Society (ICRS) grade. Radiological assessments included the Kellgren–Lawrence (KL) grading system for medial compartment osteoarthritis and hip–knee–ankle (HKA) angle.

**Results:**

Significant improvements were observed in WOMAC scores (preoperative: 57.00 (range: 44.75–63.00), postoperative: 27.50 (range: 22.25–28.75)), VAS scores (preoperative: 66.25 (range: 48.00–74.25), postoperative: 26.25 (range: 14.50–31.13)), SF-36 PCS (preoperative: 27.97 (range: 26.64–31.25), postoperative: 55.31 (range: 51.64–62.50)), and SF-36 MCS (preoperative: 41.04 (range: 29.95–50.96), postoperative: 63.18 (range: 53.83–65.16)) 2 years postoperatively (*p* = 0.002, 0.002, 0.002, and 0.020, respectively). The MFC chondral lesion demonstrated significant improvement in both lesion size (preoperative: 7.00 cm² (range: 4.38–10.50 cm²), postoperative: 0.16 cm² (range: 0.00–1.75 cm²), *p* = 0.002) and ICRS grade (preoperative: 4 (range: 4–4), postoperative: 1 (range: 1–2.25), *p* = 0.002). Additionally, the KL grade significantly decreased from 3 (range: 3–3) preoperatively to 2 (range: 2–2) postoperatively, while the HKA angle was corrected from 7.50° (range: 7.00–10.25°) preoperatively to -1.00° (range: -3.5–0.00°) postoperatively.

**Conclusions:**

hUCB-MSC implantation with HTO is an effective treatment for medial compartment osteoarthritis and varus deformities, resulting in significant improvements in cartilage regeneration and overall clinical outcomes.

**Trial registration:**

NCT04234412.

## Background

Osteoarthritis (OA) is a whole joint disease involving inflammation, fibrosis of the infrapatellar fat pad [1] and meniscal degeneration affecting the cartilage, subchondral bone, and surrounding synovial structures [2]. It is a major health issue, particularly in aging populations, with significant economic costs owing to treatments such as total knee arthroplasty [3]. The global rise in obesity and joint injuries is contributing to the rising prevalence of OA [3–5]. Approximately 14 million individuals in the United States suffer from symptomatic knee OA, with over half of these cases classified as advanced OA [4]. Silverwood et al. [6] reported that being overweight (pooled odds ratio 1.98, 95% CI 1.57–2.20) and obesity (pooled odds ratio 2.66, 95% CI 2.15–3.28) were strongly associated with the onset of knee pain in OA. Additionally, individuals with a history of joint trauma are at a substantially higher risk of developing knee OA, with odds approximately 4 to 6 times higher for those with anterior cruciate ligament or meniscal injuries compared to non-injured knees [5].

High tibial osteotomy (HTO) has been recognized as an effective surgical option for middle-aged patients with severe medial compartment OA and varus deformity [7, 8]. By shifting the weight-bearing axis from the medial to the lateral compartment, HTO can effectively preserve the knee joint and achieve favorable clinical outcomes [3, 7, 9]. Although HTO has shown excellent short- and mid-term results, long-term survival rate cannot be guaranteed [3, 7]. Hui et al. [7] reported that the probabilities of survival after HTO at 5, 10, and 15 years were 95%, 79%, and 56%, respectively. In a systematic review of 18 studies including 1296 knees, Fabbro et al. [8] demonstrated an average 10-year survival rate of 74.6%. Recently, cartilage regeneration treatment with HTO has emerged as a promising approach, demonstrating favorable clinical results and effectiveness in treating chondral lesions associated with varus deformities [10, 11]. Several studies have suggested that combining cartilage regeneration treatment with HTO can promote significant cartilage regeneration, as observed on second-look arthroscopy [10, 12]. Kahlenberg et al. [11] reported in a systematic review that the rate of conversion to total knee arthroplasty was 6.8%, with the average time from cartilage regeneration treatment with HTO to conversion ranging from 4.9 to 13.0 years. However, indications for combined surgery remain controversial.

Various cartilage regeneration techniques are used with concomitant HTO, including microfracture, bone marrow aspirate concentrate (BMAC), autologous chondrocyte implantation, autologous collageninduced chondrogenesis, and mesenchymal stem cell (MSC) therapy [10, 13]. While focal chondral lesions have been effectively treated with isolated cartilage regeneration techniques alone, large degenerative chondral lesions lead to rapid OA progression despite cartilage regeneration efforts [14, 15]. Allogenic human umbilical cord blood–derived mesenchymal stem cell (hUCB-MSC) implantation has emerged as a promising treatment for degenerative OA and varus deformity [10, 12, 16, 17]. hUCB-MSCs offer several advantages, including noninvasive collection, hypo-immunogenicity, high expansion ability, and superior cartilage repairability [18, 19]. Additionally, as an allogeneic cell source, hUCB-MSCs are produced as an off-the-shelf product, providing a sufficient supply of high-purity stem cells suitable for addressing large and diffused chondral lesions [10, 20]. However, several limitations have been found, such as poor survival, engraftment, and control of MSC chondrogenic differentiation fate in vivo [17, 21]. To improve clinical outcomes in patients with chondral lesions and varus deformity, hUCB-MSC implantation with concomitant HTO presents a promising option. However, the studies demonstrating the implantation of hUCB-MSCs with concomitant HTO are limited, and few prospective or comparative studies have been reported [10]. The purpose of this study was to evaluate the clinical outcomes before and after treatment and to assess cartilage regeneration via second-look arthroscopy. We hypothesized that the implantation of hUCB-MSCs with concomitant HTO would enhance both clinical outcomes and cartilage regeneration.

## Methods

### Participants

The study was approved by the Institutional Review Board of the instate (UC19OISI0134). The diagnosis of symptomatic medial compartment OA with varus malalignment in examined patients was confirmed through clinical symptoms, physical examination, and radiographic evaluation. The severity of OA was classified using the Kellgren–Lawrence (KL) grading system, and varus malalignment was assessed by measuring the hip-knee-ankle (HKA) angle on standing lower extremity radiographs. The inclusion criteria for this study were as follows: (1) diagnosis of symptomatic (≥ 3 months) medial compartment OA of KL Grade II or III with varus deformity (≥ 5° and < 15°), (2) age between 50 and 65 years with a body mass index (BMI) < 35 kg/m², (3) full knee flexion > 120° with < 15° flexion contracture, (4) no severe knee joint instability, defined as ≤ Grade I on physical examination (Lachman test, anterior/posterior drawer test, varus/valgus stress test), (5) no severe OA in the patellofemoral and lateral compartments on radiographs. The exclusion criteria included previous knee surgery, history of metabolic arthritis or knee joint infection, and presence of articular cartilage lesions in the lateral compartment. A total of 10 patients underwent hUCB-MSC implantation with concomitant HTO, all performed by a senior orthopedic surgeon in our prospective study. All data in this study were collected prospectively.

### Preparation of hUCB-MSCs

The study employed CARTISTEM^®^ (Medipost, Seongnam-si, Gyeonggi-do, South Korea, https://en.medi-post.co.kr/cartistem/), an off-the-shelf product designed for cartilage regeneration. This product contains 1.5 mL of hUCB-MSCs (7.5 × 10^6^ cells/vial) combined with a 4% hyaluronic acid hydrogel and was approved for cartilage regeneration by the Korea Food and Drug Administration in January 2012. The 4% lyophilized sodium hyaluronate was 60 mg per vial. The recommended therapeutic dose, based on the manufacturer’s guidelines, is 500 µL/cm^2^. The cartilage defect size was assessed using magnetic resonance imaging (MRI) before surgery to determine the precise therapeutic dose. Following the combination of hUCB-MSCs with the 4% hyaluronic acid hydrogel using a spatula, the mixture was transferred into a 5-mL syringe for implantation into the cartilage defect. The safety of the product was previously evaluated in a clinical trial conducted by Park et al. [19].

### Surgical procedure and rehabilitation

**Fig. 1:**
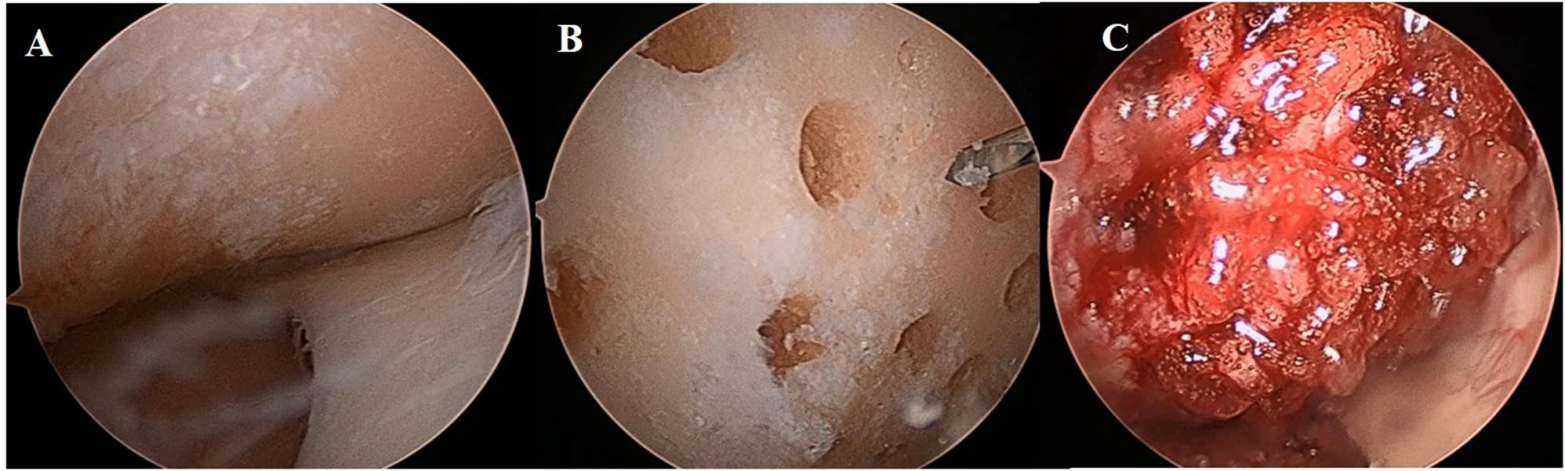
hUCB-MSC implantation in arthroscopy descripted. **A**: Medial compartment osteoarthritis in a 61-year-old woman; **B**: Multiple holes, 4 mm in diameter and 4 mm in depth, drilled using a drill bit; **C**: Human umbilical cord blood–derived mesenchymal stem cells mixed with hyaluronic acid hydrogel and implanted into the cartilage lesion. hUCB-MSC, allogenic human umbilical cord blood-derived mesenchymal stem cell

The arthroscopic procedure was performed prior to the HTO. After identifying chondral lesions in the medial femoral condyle (MFC) through arthroscopic exploration, curettes and gouges were used to debridge the surface of the chondral lesion, remove the subchondral sclerosis to expose the subchondral bone, and clearly define the lesion margins. An additional portal was created using a spinal needle to directly access the MFC chondral defect. Multiple perforations, each 4 mm in diameter and depth, were made in the chondral defect of the MFC, with a 2-mm Kirschner wire used to drill the spaces between each perforation. All debris was irrigated and the knee joint was thoroughly flushed. A mixture of hUCB-MSCs and the hyaluronic acid hydrogel was then implanted into the holes and over the articular surface. Figure 1 illustrates the surgical procedure for hUCB-MSC implantation using arthroscopy, as previously described by Song et al. [10]. Thereafter, open-wedge HTO was completed with an anatomical locking metal-block plate (Ohtofix; Ohtomedial Co. Ltd., Goyang-si, South Korea). Biplanar osteotomy was performed to align the mechanical axis laterally to approximately 62.5% of the tibial plateau. The patients were advised to engage in isometric quadriceps and hamstring exercises, while knee flexion was restricted to 90° for the first 4 weeks, progressing to complete weight-bearing at week 6. Second-look arthroscopy with hardware removal was performed 2 years after the primary surgery.

### Clinical outcomes

The Western Ontario and McMaster Universities Osteoarthritis Index (WOMAC) [22] and visual analog scale (VAS) were used to assess clinical outcomes by comparing the preoperative state with states observed 6 months, 1 year, and 2 years postoperatively. Additionally, the 36-Item Short-Form Survey (SF-36) was used to evaluate clinical outcomes based on the Physical Component Summary (PCS) and Mental Component Summary (MCS) [23].

### Radiologic and arthroscopic evaluations

**Fig. 2:**
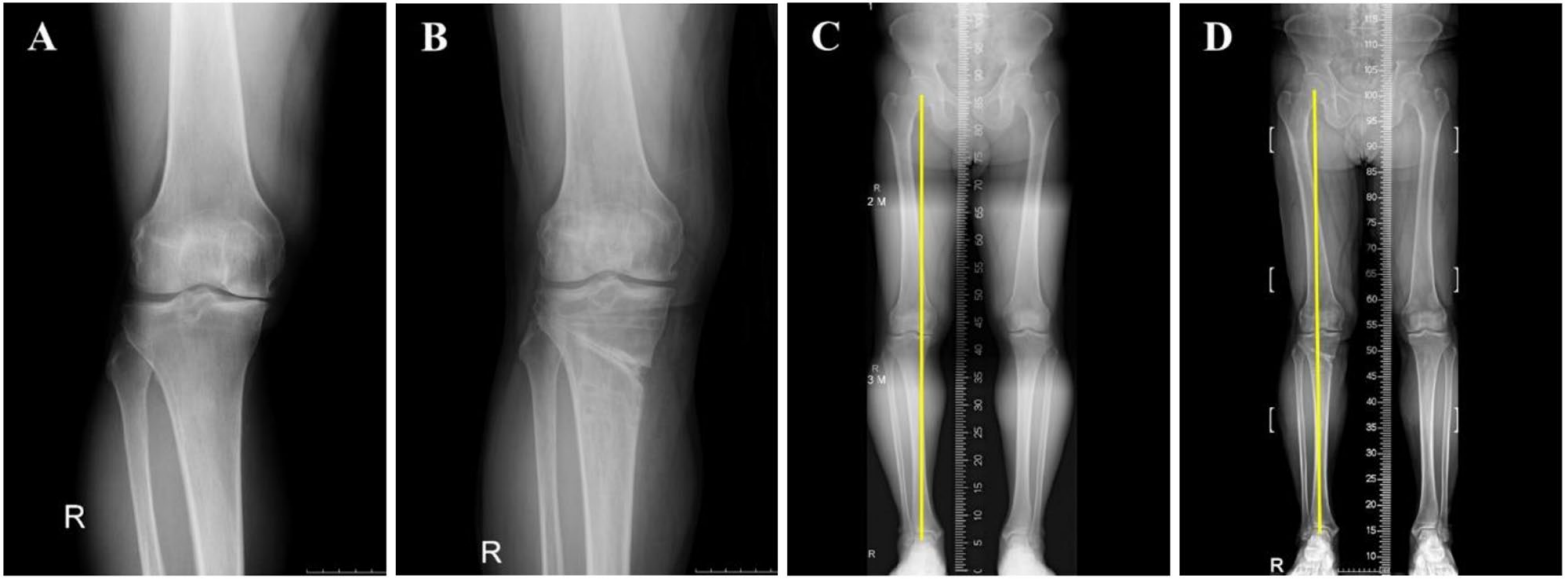
Comparison between preoperative and 2-year postoperative states in a 59-year-old man who underwent HTO on the right knee. **A**: preoperative state with KL grade III; **B**: 2-year postoperative state after hardware removal with KL grade II; **C**: preoperative state with HKA 5° angle; **D**: postoperative state with HKA − 1.5° angle. KL, Kellgren–Lawrence grading; HKA, Hip–knee–ankle; HTO, High tibial osteotomy

**Fig. 3:**
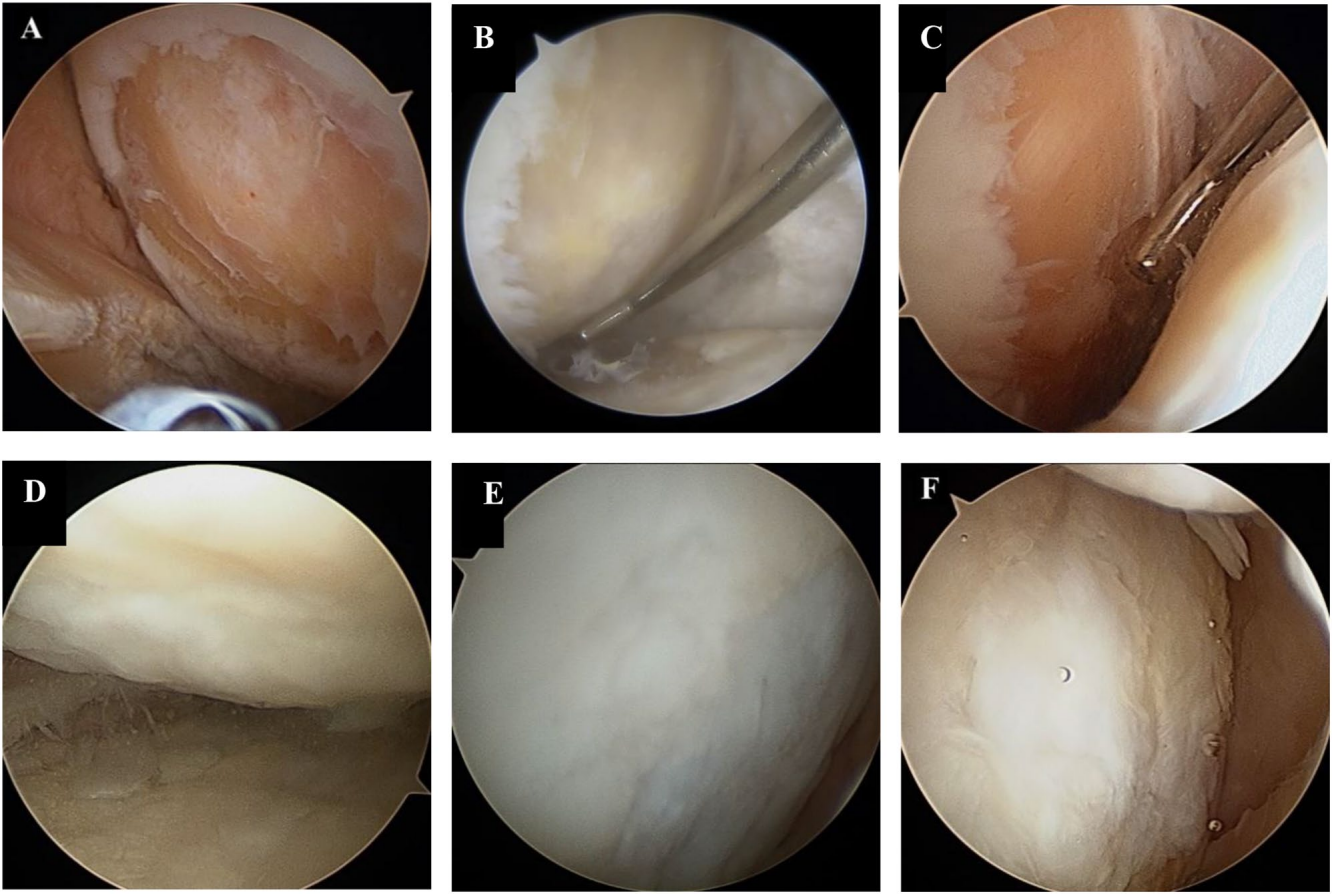
Comparison between preoperative and second-look arthroscopy. (**A**, **D**) Chondral lesion of MFC in a 60-year-old male patient preoperatively (**A**) and 2-year postoperatively (**D**). (**B**, **E**) Chondral lesion of MFC in a 59-year-old male patient preoperatively (**B**) and 2-year postoperatively (**E**). (**C**, **F**) Chondral lesion of MFC in a 57-year-old female patient preoperatively (**C**) and 2-year postoperatively (**F**). MFC, medial femoral condyle

The HKA and KL grading were compared between preoperative and 2-year postoperative states to assess coronal alignment. The HKA angle was measured using standing lower extremity radiographs by drawing a line from the center of the femoral head through the midpoint between the tibial intercondylar eminences, then extending to the center of the talus, to assess alignment. KL Grading was determined using standing anteroposterior, lateral, and Rosenberg radiographs. Figure 2 shows a comparison of the HKA angles and KL grades between the preoperative and 2-year postoperative periods. Arthroscopic evaluations were conducted both preoperatively and 2 years postoperatively using the International Cartilage Repair Society (ICRS) grading system to assess and compare cartilage status [24]. In this grading system, grade 1 cartilage defects refer to superficial chondral lesions such as superficial fissures and cracks; grade 2 defects refer to lesions that extend to less than 50% of the cartilage depth; grade 3 defects are characterized by extensions that penetrate more than 50% of the cartilage depth without reaching the subchondral bone; and grade 4 defects involve the subchondral bone [24]. Furthermore, chondral lesion size was evaluated both preoperatively and postoperatively. Figure 3 shows a comparison of chondral lesions between the preoperative and 2-year postoperative periods.

### Statistical analysis

Statistical analyses were performed using the Statistical Package for Social Sciences version 29 (IBM Corp., Armonk, NY, USA), with the significance threshold set at *p* < 0.05. The results are reported as mean ± standard deviation or number (percentage). For skewed variables, the Wilcoxon signed-rank test was used to analyze differences between the preoperative and 2-year postoperative states for clinical outcomes, radiological assessments, and arthroscopic cartilage evaluations. Additionally, Friedman’s test was used to evaluate differences in clinical outcomes across the preoperative and 6-month, 1-year, and 2-year postoperative states.

## Results

**Fig. 4:**
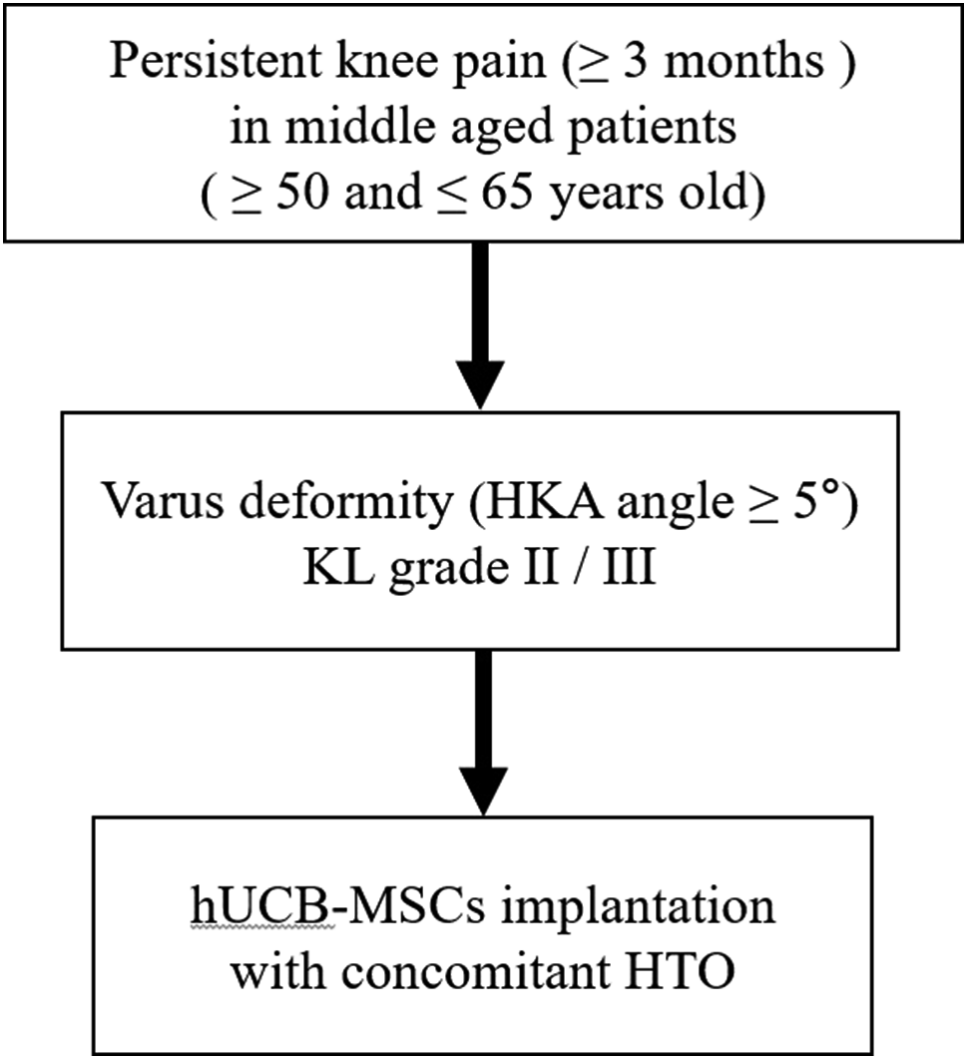
Flow chart of the study. KL, Kellgren–Lawrence grading; HKA, Hip–knee–ankle; HTO, High tibial osteotomy; hUCB-MSC, allogenic human umbilical cord blood-derived mesenchymal stem cell

Figure 4 presents the study flowchart. In this study, 10 patients with a median age of 58.50 (range: 57.00–60.00) years were included; 7 (70%) were female and 3 (30%) were male patients. The average body mass index (BMI) was 27.81 (range: 24.42–32.24) kg/m^2^, and the mean HKA angle for measuring varus deformity was 7.50 (range: 7.00–10.25)°. Table 1 presents patient demographics.

**Table 1:** Basic characteristics

Sex, male/female (*n*/*n*)	3 / 7
Age (years)	58.50 [57.00–60.00]
Side of involvement, right/left (n / n)	6 / 4
BMI (kg/cm^2^)	27.81 [24.42–32.24]
Lesion size (cm^2^)	7.00 [4.38–10.50]
HKA angle (°)	7.50 [7.00–10.25]
ICRS grade (n)	4 [4–4]
KL grade (n)	3 [3–3]
Surgery, Partial meniscectomy / Root repair (n/n)	5 / 5
WOMAC	57.00 [44.75–63.00]
VAS score	66.25 [42.25–75.50]
SF-36 PCS	27.97 [25.39–37.34]
SF-36 MCS	41.04 [26.33–53.98]

BMI, Body mass index; HKA, Hip–knee–ankle angle; ICRS, International Cartilage Repair Society; KL, Kellgren–Lawrence grading system; SF-36 MCS, 36-Item Short-Form Mental Component Summary; SF-36 PCS, 36-Item Short-Form Physical Component Summary; WOMAC, Western Ontario and McMaster Universities Osteoarthritis Index; VAS, Visual analog scale

In arthroscopic evaluations, the ICRS grade significantly improved from grade 4 preoperatively to 1 (range: 1–2.25) postoperatively (*p* = 0.002) as confirmed on second-look arthroscopy at 2 years postoperatively. The size of the chondral lesion significantly decreased from 7.00 (range: 4.38–10.50) cm^2^ preoperatively to 0.16 (range: 0.00–1.75) cm^2^ postoperatively. Radiologic assessments showed a significant improvement in the KL grade from grade 3 (range: 3–3) preoperatively to 2 (range: 2–2) at 2 years postoperatively. The HKA angle demonstrated a significant adjustment from 7.50 (range: 7.00–10.25)° to -1.00 (range: -3.5–0.00)°. Table 2 presents the arthroscopic and radiological evaluations.

**Table 2:** Comparison of preoperative and 2-year postoperative statuses based on arthroscopic and radiologic evaluations

	Preoperative (*n* = 10)	Postoperative (*n* = 10)	Difference	*p*
Lesion size (cm^2^)	7.00 [4.38–10.50]	0.16 [0.00–1.75]	6.00 [4.38–7.81]	0.002^a^
HKA angle (°)	7.50 [7.00–10.25]	-1.00 [-3.5–0.00]	10.50 [8.5–11.25]	0.002^a^
ICRS grade (n)	4 [4–4]	1 [1–2.25]	3 [1.75–3]	0.002^a^
KL grade (n)	3 [3–3]	2 [2–2]	1 [1–1]	0.004^a^

^a^Wilcoxon signed-rank test: Preoperatively vs. 2 years postoperatively

HKA, Hip–knee–ankle; ICRS, International Cartilage Repair Society; KL, Kellgren–Lawrence grading system

Based on Friedman’s test, the preoperative, 6-month postoperative, 1-year postoperative, and 2-year postoperative WOMAC, VAS, and SF-36 PCS and MCS scores were evaluated and demonstrated significant improvement (WOMAC: *p* < 0.001, VAS: *p* < 0.001, SF-36: *p* < 0.001, SF-36 MCS: *p* < 0.014). VAS (preoperative: 66.25 (range: 42.25–75.50), 6-month postoperative: 38.75 (range: 25.50–58.50), 1-year postoperative: 38.00 (range: 16.63–48.50), 2-year postoperative: 26.25 (range: 13.63–32.38)) and SF-36 PCS scores (preoperative: 27.97 (range: 25.39–37.34), 6-month postoperative: 43.75 (range: 29.84–48.98), 1-year postoperative: 48.91 (range: 35.55–59.29), 2-year postoperative: 55.31 (range: 49.38–63.75)) showed significant improvement over the follow-up period. WOMAC and SF-36 MCS scores showed no significant improvement 6 months postoperatively (WOMAC: 47.50 (range: 35.50–54.50), SF-36 MCS: 55.94 (range: 38.13–59.43)) compared with preoperative scores (WOMAC: 57.00 (range: 44.75–63.00), SF-36 MCS: 41.04 (range: 26.33–53.98)). However, significant improvements were confirmed at both 1 year (WOMAC: 36.0 (range: 28.50–47.75), SF-36 MCS: 54.06 (range: 40.65–66.98)) and 2 years (WOMAC: 27.50 (range: 21.50–29.50), SF-36 MCS: 63.18 (range: 53.67–66.33)) postoperatively. No reoperations or complications were observed. Table 3 presents the clinical outcomes.

**Table 3:** Comparison between preoperative and postoperative clinical outcomes

-	WOMAC	*p*	VAS	*p*
Preoperative 6-month postoperative 1-year postoperative 2-years postoperative	57.00 [44.75–63.00] 47.50 [35.50–54.50] 36.0 [28.50–47.75] 27.50 [21.50–29.50]	< 0.001^a^ 0.189^b^ 0.004^c^ 0.002^d^	66.25 [42.25–75.50] 38.75 [25.50–58.50] 38.00 [16.63–48.50] 26.25 [13.63–32.38]	< 0.001^a^ 0.002^b^ 0.002^c^ 0.002^d^
-	SF-36 PCS	*p*	SF-36 MCS	*p*
Preoperative 6-month postoperative 1-year postoperative 2-years postoperative	27.97 [25.39–37.34] 43.75 [29.84–48.98] 48.91 [35.55–59.29] 55.31 [49.38–63.75]	< 0.001^a^ 0.037^b^ 0.002^c^ 0.002^d^	41.04 [26.33–53.98] 55.94 [38.13–59.43] 54.06 [40.65–66.98] 63.18 [53.67–66.33]	0.014^a^ 0.064^b^ 0.049^c^ 0.020^d^

^a^Friedman test: Preoperatively vs. 6 months postoperatively vs. 1 year postoperatively vs. 2 years postoperatively

^b^Wilcoxon signed-rank test: Preoperatively vs. 6 months postoperatively

^c^Wilcoxon signed-rank test: Preoperatively vs. 1 year postoperatively

^d^Wilcoxon signed-rank test: Preoperatively vs. 2 years postoperatively

SF-36 MCS, 36-Item Short-Form Mental Component Summary; SF-36 PCS, 36-Item Short-Form Physical Component Summary; WOMAC: Western Ontario and McMaster Universities Osteoarthritis Index; VAS: Visual analog scale

## Discussion

The most significant finding of this study was that the clinical outcomes of hUCB-MSC implantation with concomitant HTO were excellent, with prominent cartilage regeneration observed on second-look arthroscopy. The patients included in our study were relatively older, with a median age of 58.50 (range: 57.00–60.00) years. The BMI was 27.81 (range: 24.42–32.24) kg/m², indicating overweight patients, and approximately 30% of the total participants had a BMI ≥ 30 kg/m². The mean size of the preoperative chondral lesions was 7.00 (range: 4.38–10.50) cm², with 90% of all participants having chondral lesions > 4 cm². Therefore, hUCB-MSC implantation with concomitant HTO proved to be effective even in patients with large-sized chondral lesions (≥ 4 cm^2^) and varus deformity (HKA ≥ 5°) as well as in middle-aged overweight patients.

All clinical outcomes significantly improved in this study. The statistical difference indicated by Friedman’s test suggests that the clinical outcomes at the preoperative and 6-month, 1-year, and 2-year postoperative stages showed significant gradual improvement over the follow-up period. In this study, no significant improvement was observed in WOMAC and SF-36 MCS scores at the 6-month postoperative stage compared with the preoperative stage. However, at the 1-year and 2-year postoperative stages, all clinical evaluations showed significant improvements compared with the preoperative stage. All clinical evaluations at the 2-year postoperative stage showed significant improvement compared with the preoperative stage, confirming that the short-term follow-up results were positive.

For radiologic assessments in this study, KL grade 3 patients showed a significant improvement to grade 2 at 2 years postoperatively. This improvement is likely owing to corrected alignment and improved joint space narrowing after surgery. Several studies have already demonstrated medial joint space widening following HTO, reporting an improvement in the OA grade [25, 26]. Preoperative joint space narrowing and the joint line convergence angle significantly impact clinical satisfaction in patients following HTO and are also considered risk factors for OA progression [27, 28]. However, although an improvement in the KL grade may be observed, it is not necessarily associated with cartilage regeneration. Several authors, in retrospective studies of patients who underwent isolated HTO, reported that medial joint space widening showed a significant correlation with the weight-bearing line ratio but did not exhibit a significant correlation with cartilage regeneration [29, 30].

HTO has been an alternative treatment for middle-aged patients with severe OA in the medial compartment and varus malalignment (≥ 5°), aiming to prevent further OA degeneration and progression to total knee replacement. The long-term clinical outcomes have already been well established [8, 31, 32]. Constatin et al. [31] reported that the 5-, 10-, and 20-year HTO survival rates of 88%, 77%, and 44% for HTO, respectively, with favorable candidates (age < 55, BMI < 30, and WOMAC pain score > 45) achieving a survival rate of 62% at 20 years. Mechanical alignment and ligament stability have been demonstrated to influence clinical outcomes and survival rate in the long-term follow-up [32, 33]. The load distribution after HTO shifting weight-bearing axis to the lateral compartment lead to increase contact pressure of lateral compartment, which may affect MFC articular cartilage [34]. However, simple HTO alone has not shown significant superior advantage of MFC cartilage regeneration compared to simple cartilage repair in second-look cartilage status in short-term follow-up studies, although postoperative MFC cartilage has demonstrated significant improvement [35, 36]. Kim et al. [35] observed that, following HTO without additional cartilage repair, chondral lesions in the MFC and medial tibial plateau improved in 54 knees (51.9%) and 36 knees (34.6%), respectively (*n* = 104, mean age: 56.3 ± 5.4 years, mean follow-up 25.0 ± 5.8 months, mean HKA: 6.0 ± 2.2°). Jung et al. [36] reported that chondral lesions in the MFC exhibited grade II cartilage status (white scattering with fibrocartilage, partial coverage with fibrocartilage, or even coverage with fibrocartilage), in 94% of patients undergoing HTO (*n* = 31, mean age: 58.6 ± 6.9 years) and 100% of patients undergoing HTO with subchondral drilling (*n* = 30, mean age: 61.5 ± 7.5 years), with no significant difference between the two groups (*p* = 0.492). Additionally, several comparative studies have demonstrated that simple cartilage repair methods show improved cartilage regeneration in the short-term follow-up compared to the microfracture with HTO group [18, 37].

Cartilage repair techniques are considered controversial to influence clinically in long-term follow-up. Unfortunately, many studies have failed to demonstrate cartilage improvement in long-term follow-up with isolated cartilage repair alone. Recent studies have been reported that HTO with ACI improve clinically in the long-term follow-up [38–40]; however, it has been limited to the patients with small to medium chondral lesion or relatively young patients. Cartilage improvement in large-sized, degenerative chondral lesions of the medial compartment is highly challenging [41]. Throughout our study, hUCB-MSC implantation with concomitant HTO may be alternative treatment that can influence cartilage status and clinical outcomes compared to conventional HTO treatment or simple cartilage repair.

In this study, all patients showed improvement on second-look arthroscopy 2 years postoperatively compared with preoperative values without complications. A notable point in our study is that cartilage regeneration at the 2-year follow-up is considerably better compared to other MSC implantation studies [12, 18, 42]. These results demonstrate that hUCB-MSC implantation with concomitant HTO is an effective treatment for patients with large chondral lesions and varus deformities. Jung et al. [18] conducted a prospective comparative study with a one-year follow-up, evaluating the outcomes of hUCB-MSC implantation without osteotomy versus microdrilling with HTO. The mean ICRS grade following hUCB-MSC implantation was 2.2 ± 0.68 grade (*n* = 15, mean age: 59.2 ± 5.4 years, preoperative MFC chondral lesion: 7.2 ± 2.0 mm^2^). In contrast, the mean ICRS grade after microdrilling with HTO was 2.5 ± 0.97 grade (*n* = 10, mean age: 57.9 ± 3.7 years, preoperative MFC chondral lesion: 5.2 ± 2.1 mm^2^). Song et al. [10] reported a retrospective study in which the ICRS grade after hUCB-MSC implantation combined with HTO was 1.54 ± 0.70 grade (*n* = 125, mean age: 58.3 ± 6.8 years, preoperative MFC chondral lesion: 6.9 ± 2.0 mm^2^, mean HKA: 7.6 ± 2.4°). In our study, the ICRS grade after hUCB-MSC implantation combined with HTO was 1 (range: 1–2.25). Compared to the methods applied in the study by Jung et al. [18] with simple hUCB-MSC implantation and the study by Song et al. [10], the outcomes of this study can be considered superior. Therefore, the results of our study suggest that hUCB-MSC implantation enhances cartilage regeneration more effectively when combined with HTO, offering a promising therapeutic strategy for patients with medial compartment OA and varus deformity.

There is a need to investigate the reasons for the superior outcomes of hUCB-MSC implantation both clinically and biologically. Lee et al. [12] reported that ICRS grading at second-look arthroscopy was better in patients who underwent hUCB-MSC implantation (*n* = 32, mean age: 58.1 ± 3.6 years, mean MFC chondral lesion: 7.0 ± 1.9 mm^2^, ICRS grading: from 3.9 to 2.0) combined with HTO compared to those who received BMAC with HTO (*n* = 42, mean age: 60.7 ± 4.1 years, mean MFC chondral lesion: 6.5 ± 2.9 mm^2^, ICRS grading: from 3.9 to 2.8), even though no significant differences were observed in clinical and radiologic outcomes between the two groups (mean follow-up: 18.7 ± 4.6 months). Similarly, Park et al. [42] conducted a systematic review and meta-analysis, concluding that hUCB-MSCs combined with HTO (*n* = 330, mean age: 57.8 ± 5.4 years) were more effective in articular cartilage regeneration than BMAC augmentation with HTO (*n* = 169, mean age: 57.7 ± 7.3 years), with greater improvement in articular cartilage regeneration in patients treated with hUCB-MSCs (SMD, 4.18; 95% CI, 3.61–4.75) compared to those treated with BMAC (SMD, 1.81; 95% CI, 1.10–2.53; *p* < 0.001). These clinical findings suggest that hUCB-MSCs may have a higher capacity for multilineage differentiation compared to bone marrow-derived mesenchymal stem cells (BM-MSCs). However, there are few comparative studies evaluating cartilage regeneration in knee OA between hUCB-MSCs and adipose tissue-derived mesenchymal stem cells (AD-MSCs) implantations. Koh et al. [43] reported 87.5% of elderly patients (14/16, mean age: 70.3 [65–80] years) showed improvement or maintained cartilage status in second-look arthroscopy at least two years after AD-MSCs implantation however, no control group was observed. Since there are limited comparative studies among different types of MSCs and a lack of long-term clinical studies, further research is necessary for a more comprehensive comparison and analysis.

Biologically, hUCB-MSCs can differentiate into various musculoskeletal lineages, including cartilage, bone, and adipose tissue, and have shown particularly strong chondrogenic differentiation potential, which is beneficial for cartilage repair [44, 45]. hUCB-MSCs are considered more primitive compared to other MSC sources, contributing to lower immunogenicity and allowing for a greater degree of HLA mismatch in transplantation [44, 45]. Oh et al. [46] compared AD-MSCs, BM-MSCs, and UCB-MSCs in a rat model of pulmonary hypertension and found that UCB-MSCs had the strongest effect in reducing inflammation and improving right ventricular function. The chondrogenic ability of AD-MSCs is generally considered inferior to that of BM-MSCs, but AD-MSCs possess a greater proliferative capacity. However, Ma et al. [47] concluded that amniotic membrane-derived MSCs, umbilical cord-derived MSCs, and chorionic plate-derived MSCs exhibited similar chondrogenic potential, as indicated by comparable expression levels of DCN, COMP, and COL2A1 in real-time PCR analysis. MSCs have emerged as a promising option for cartilage repair due to their ability to differentiate into chondrocytes and their immunomodulatory effects. However, they frequently result in suboptimal tissue composition and mechanical performance, leading to limitations in long-term efficacy. Additionally, challenges related to donor selection, the invasiveness of procedures, and variability in tissue responses must be addressed to improve clinical outcomes in cartilage regeneration [48].

Although this study presents important findings, it has some limitations. First, this study had a relatively short follow-up period and small sample size. Mid- or long-term follow-up with a large patient study will provide accurate results for the efficacy of hUCB-MSC implantation with concomitant HTO. Second, there was no control group in this study, and the efficacy of hUCB-MSC implantation could not be definitively established. A comparative analysis with control groups, such as isolated hUCB-MSC implantation or isolated HTO, would provide stronger evidence for the effectiveness of hUCB-MSC implantation with concomitant HTO. Third, while MRI is a non-invasive tool that can assist in evaluating cartilage repair, we used second-look arthroscopy as it allows for direct visualization and grading of cartilage quality, which is considered the gold standard for assessing cartilage regeneration. Arthroscopic evaluation provides a more detailed assessment of the repaired tissue’s structural integrity, surface smoothness, and integration with native cartilage, which MRI may not fully capture. However, future studies incorporating MRI-based assessments, such as the Magnetic Resonance Observation of Cartilage Repair Tissue 2.0 knee score, could further complement arthroscopic findings and offer a comprehensive evaluation of cartilage regeneration from multiple perspectives.

## Conclusion

The implantation of hUCB-MSCs with concomitant HTO is an effective treatment for patients with medial compartment OA and varus deformity, resulting in improved cartilage regeneration. This study suggests that hUCB-MSC implantation with concomitant HTO is an appropriate treatment option, even for middle-aged patients with large chondral lesions, overweight BMI, and varus deformity.

## Data Availability

All additional files are included in the manuscript.

## Abbreviations

AD: MSC–adipose tissue–derived mesenchymal stem cell
BMI: Body mass index
BMAC: Bone marrow aspirate concentrate
BM: MSC–bone marrow–derived mesenchymal stem cell
HKA: Hip–knee–ankle
hUCB: MSC–human umbilical cord blood–derived mesenchymal stem cell
HTO: High tibial osteotomy
ICRS: International Cartilage Repair Society
KL: Kellgren–Lawrence
MCS: Mental Component Summary
MFC: Medial femoral condyle
MRI: Magnetic resonance imaging
OA: Osteoarthritis
PCS: Physical Component Summary
SF: 36–36–Item Short–Form Survey
VAS: Visual analog scale
WOMAC: Western Ontario and McMaster Universities Osteoarthritis Index
